# Morphology and Thermodynamic Study of a Novel Composite Membrane from Waste Polystyrene/Slag: Experimental Investigation

**DOI:** 10.1021/acsomega.4c00671

**Published:** 2024-05-21

**Authors:** Salma Tarek Ghaly, Usama Nour Eldemerdash, A. H. El-Shazly

**Affiliations:** † Chemical and Petrochemical Engineering Department, Egypt-Japan University of Science and Technology, New Borg AL Arab City, 21934 Alexandria, Egypt; ‡ Central Metallurgical Research and Development Institute (CMRDI), P.O. Box 87 Helwan, 11421 Cairo, Egypt; § Benha Faculty of Engineering, Benha University, 13511 Qaliobiya, Egypt; ∥ Chemical Engineering Department, Faculty of Engineering, Alexandria University, 5424041 Alexandria, Egypt

## Abstract

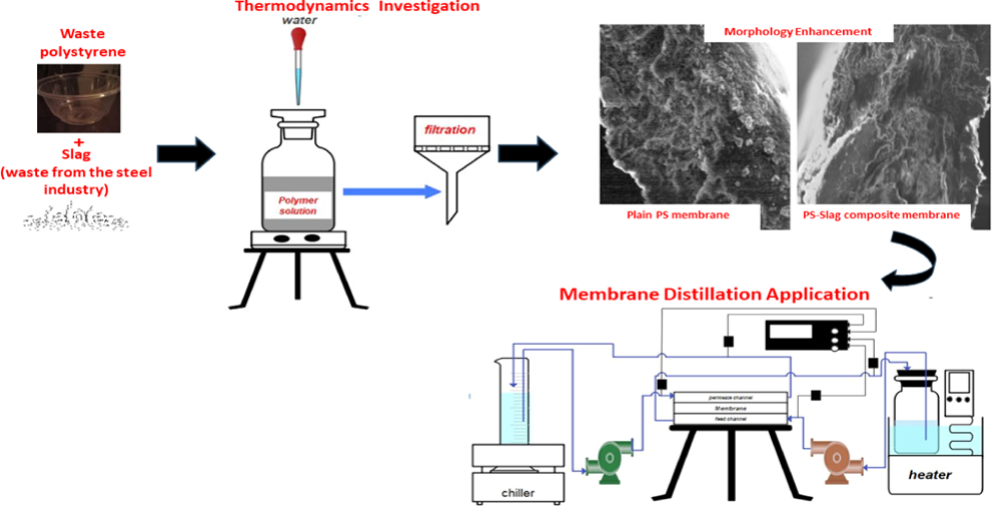

The development of the membrane surface and cross-sectional morphology is pivotal in influencing the effectiveness of membrane separation. In this study, evaluating the separation rates between the solvent and nonsolvent in the casting solution and the related thermodynamic alteration analysis were illustrated. Additionally, the rheological variations were determined by measuring the viscosity of the resulting dope solutions, providing an initial estimation of the phase separation kinetics. Asymmetric polystyrene (PS)/slag composite membrane, incorporating slag waste as an inorganic additive, was developed. Dimethylformamide (DMF) was utilized as the solvent, and sodium dodecyl sulfate (SDS) was employed as an anionic surfactant to facilitate the casting process. A tertiary system diagram approach involving waste PS, DMF, and water introducing slag as an inorganic additive and SDS as a surfactant was attained to promote the separation of the solvent and nonsolvent in the casting solution. These novel composite mixtures exhibited increased thermodynamic instability within the coagulation bath, facilitating the rapid separation of solid membranes from the dope solutions and forming composite membranes with significantly increased porosity (exceeding a 20% increase) compared to that of plain waste materials. The composite membrane characteristics were assessed with the widely used poly(vinylidene difluoride) (PVDF) membrane, showing comparative features and performance when tested on a membrane distillation (MD) cell; it gave a flux of 1 kg/m^2^·h. These promising characteristics positioned this novel PS/slag composite membrane as a candidate for various water-related applications.

## Introduction

Plastic products, renowned for their versatility and broad applications, have led to significant industry growth with over 8300 million metric tons produced globally. The substantial global population growth coupled with a desire for improved living standards has led to a significant surge in polymer (especially plastics) consumption. Yearly, the usage of plastic materials has soared from about 5 million tons in the 1950s to nearly 100 million tons today. Unfortunately, the short lifespan of plastic products, with roughly 40% having a duration of less than one month, resulted in a substantial waste stream, contributing to severe environmental issues.^1^ Egypt generates approximately 60 million tons of solid waste annually, with plastics constituting 3–12% of this total.^2^ On a global scale, 79% of plastic waste is disposed of in landfills or the environment; 12% is incinerated, and only 9% is recycled. The production of municipal solid waste (MSW) worldwide has reached 1.3 billion tons per year, with plastic solid waste (PSW) comprising approximately 8–13% in developing and emerging countries.^3^ However, due to regulatory constraints, increasing costs, and the poor biodegradability of common polymers, landfill disposal is less favored. Therefore, recycling presents a more viable solution. However, this surge in nonbiodegradable plastic waste, primarily derived from nonrenewable sources, poses environmental challenges, contributing to widespread pollution.^4^

Polystyrene (PS) ranks as the fourth most produced thermoplastic based on volume. Approximately 21 million tons of PS was manufactured globally in 2013.^5^ Its utilization is prominent in various major markets, with packaging, consumer/institutional goods, electrical/electronic goods, building/construction, furniture, industrial/machinery, and transportation being the primary sectors in order of consumption.^6^ In recent years, there has been limited focus on styrenic polymers, including high-impact polystyrene, despite accounting for approximately 12% of the total thermoplastics’ consumption in Europe in 2003.^7^ The disposable and lightweight nature of various PS-derived materials has led to environmental accumulation as nondegradable waste.^8^ Regrettably, like many other plastics, the primary purpose of utilizing PS has been for a single use. Polystyrene is used in both solid and expanded forms, offering recyclability opportunities.

Moreover, the steelmaking industry generates significant waste, including sludge and slag.^9,10^

Various types of slag, such as electric arc furnace slag (EAF), blast furnace slag (BF), ladle furnace slag (LF), and oxygen furnace slag (BOF), are produced based on the steel manufacturing process.^11,12^ Steel slag, rich in CaO, Fe_2_O_3_, SiO_2_, and other components, serves as a flux material, replacing limestone.^13^ Its applications range from railway ballast to metal recovery, but annual global production exceeds 50 million tons, necessitating proper treatment and disposal to combat environmental pollution.^14,15^

The widespread application of membrane technology is well-established, particularly in water treatment, desalination, and industrial sectors owing to its reliability and operational ease. Polymeric membranes, favored for their cost-effective manufacturing, are primarily produced through phase inversion, including recycled materials for sustainable membrane development. These waste-derived membranes demonstrate separation efficiency in part with commercial alternatives. The use of recycled high-impact polystyrene (HIPS) from packaging to create ultrafiltration (UF) membranes resulted in efficient removal of phenolic compounds (40%) and significant color removal (75%), comparable to commercial polyethersulfone (PES) and cellulose acetate (CA) membranes.^4,16^ This shift toward recycling aligns with the UN’s Sustainable Development Goals (SDGs), specifically goal 12, which advocates sustainable consumption and production practices. Plastics like PET, HDPE, PVC, and HIPS, known for their lightweight, strength, and chemical resistance, simplify membrane production. Their hydrophobic nature suits membrane distillation by creating a gas–liquid interface to prevent liquid transfer.

Prior research explored the integration of iron oxide with polystyrene in membrane technology for structural improvement. These particles, such as Fe_3_O_4_, replaced ethylenediamine, strengthening the membrane’s structure by connecting graphene plates. Combining them with polystyrene enhanced hydrophobicity, promoting water resistance and oil absorption. The magnetic properties enabled efficient recovery using magnets, enhancing the structural strength, hydrophobicity, and magnetism. Another study introduced a vacuum membrane distillation (VMD) technique, utilizing a ’self-heating’ membrane mechanism with iron oxide-carbon nanotubes. This novel approach lowered energy consumption compared with traditional VMD, demonstrating the potential of iron nanoparticles for induction heating. Additionally, a composite material was synthesized using sulfonated waste expanded polystyrene (SWPS) and iron(II) oxide nanoparticles (FeO-NPs) to efficiently degrade indigo carmine dye.^17−19^

This study introduced the use of a nonsolvent-induced phase inversion casting technique to create a novel PS/Slag/SDS composite membrane. The membrane was developed using HIPS and steel slag along with an anionic surfactant, sodium dodecyl sulfate (SDS). This study assesses the thermodynamic stability of this tertiary system with additives for prediction of crafting microporous membranes with enhanced membrane’s properties. The thermodynamic characteristics, represented by the binodal line and miscibility gap, were observed to predict the demixing process. Viscosity measurements were conducted to anticipate the precipitation rate, which influenced the membrane morphology. The membranes produced were subsequently analyzed for morphology, porosity, and contact angle. Furthermore, the composite membrane was tested on our customized lab-scale direct contact membrane distillation cell for performance testing.

## Materials and Methods

### Materials

The membrane’s polymer matrix was derived from locally sourced waste plastic of PS, obtained from the Egyptian market. An electric arc furnace (EAF) slag from Ezz Steel in Egypt was used in this study. Dimethylformamide (DMF) was procured from Fisher in the United States (≥99% laboratory reagent grade), sodium dodecyl sulfate (SDS) was purchased from Sigma-Aldrich (ACS reagent, ≥99.0%), and bi-distilled water was employed for preparing the solutions. Before usage, the cups were sanitized, dried, and chopped into square fragments (2 mm^2^). The steel slag was subjected to ball milling using a planetary ball mill with a grinding jar volume of 50 mL. The grain size of the slag was smaller than 3 mm, and the milling process was conducted at room temperature with a grinding speed of 360 rpm for a duration of 30 min until passing through a screen sieve of size (106 μm). A poly(vinylidene difluoride) (PVDF) commercial sheet membrane (Amersham Hybond, P western plotting membranes, catalog no. 10600057) was used for comparing the properties and testing performance.

### Methods

#### Raw Material Characterization

Waste polystyrene was characterized using Fourier-transform IR spectroscopy (FTIR) (Vertex 70, Bruker Scientific Instruments, Baden-Württemberg, Germany) at ambient circumstances with a wave range of 4000–400 cm^–1^ for structure and functional groups. Besides, X-ray diffraction (XRD) using a Shimadzu-6100 (Japan) was also implemented for phase determination. For slag chemical composition, X-ray fluorescence (XRF) was performed using a Rigaku NEX CG EDXRF (Japan). A Zetasizer analyzer (Malvern Zetasizer Nano Series, Germany) was used for slag particle size determination.

The plain PS was studied for concentration range (15–35%) as less conc. (10 wt %) would not form a membrane and would be destructed in a coagulation bath. In addition, more concentration will lead to a very viscous dope solution that is difficult to cast.^20−22^ Both the slag and SDS addition range were predetermined according to the highest range recorded in the literature^17−19,23^ in the range (0.1–1.5 wt %) with respect to the weight of solution and the weight of DMF, respectively.

#### Membrane Preparation

The choice for membrane fabrication has leaned toward the nonsolvent-induced phase separation method (NIPS) due to its preference for less intricate preparation methods and the need for fewer sophisticated instruments, making it easily reproducible and scalable. The NIPS technique transforms a polymer from a liquid state to a solid state through immersion precipitation. Membrane formation involves phase separation, where a homogeneous polymer solution transitions into a two-phase system: a solid-polymer-rich phase forming the membrane structure and a liquid-polymer-poor phase forming the membrane pores. This process is induced by introducing a precipitant into the polymer solution, causing an unstable state with increased polymer activity and directed polymer transport. Phase separation initiates at the surface of the film due to the concentration of the precipitant reaching a critical level, while the polymer concentration in the interior remains below the threshold for phase separation. This leads to a net movement of polymer toward the surface, creating a concentrated skin layer that inhibits further flux of precipitant and solvent. Consequently, the skin acts as a barrier for precipitant transport, resulting in less steep concentration profiles within the casting solution. As the skin forms, diffusion of the precipitant into the sublayer slows, creating areas of varying polymer concentrations that serve as nucleation centers for polymer precipitation. These areas, scattered throughout the film, contribute to the random distribution of the polymer structure during precipitation.^21^ The inclusion of inorganic additives into the polymer dope solution is a promising strategy for enhancing the membrane performance in membrane distillation (MD). It could modify the membrane structure by enhancing crucial membrane properties, such as porosity, hydrophobicity, pore size, and others.^24,25^

**Figure 1:**
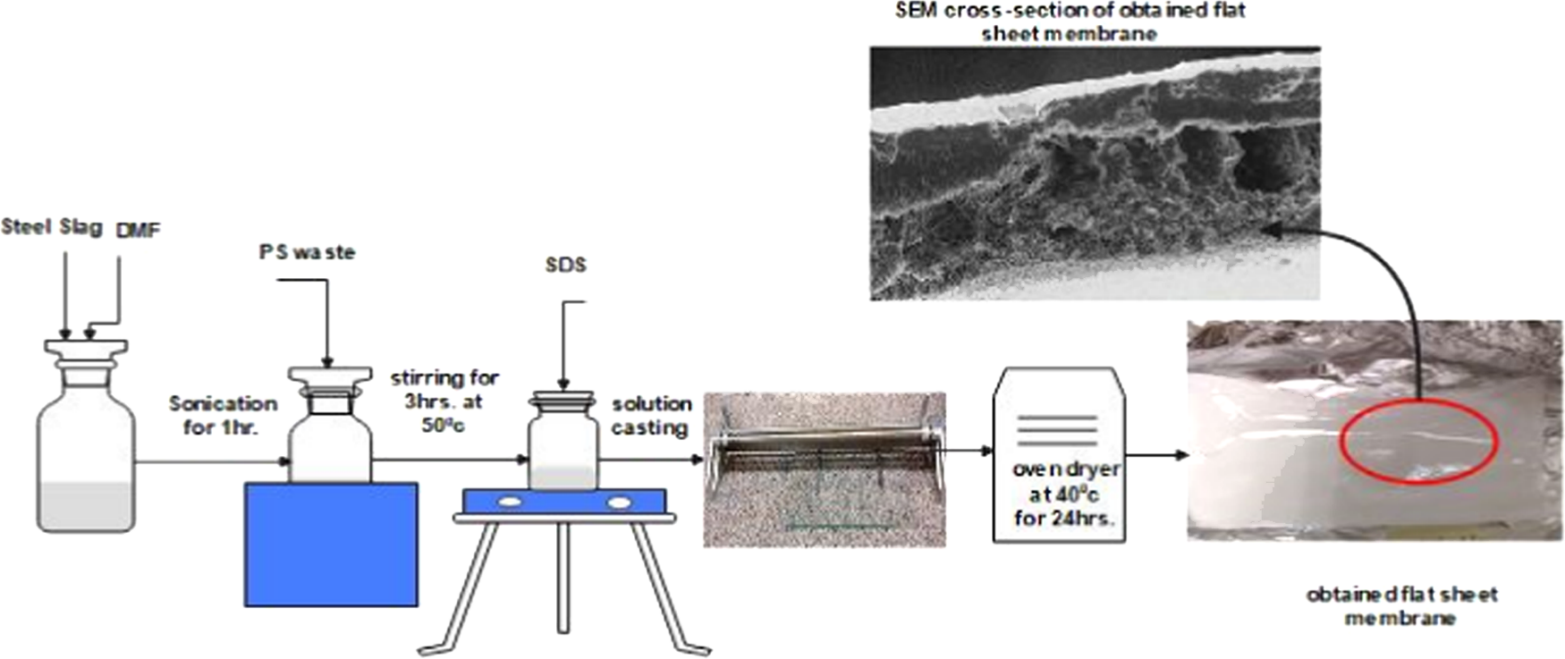
Schematic diagram of dope solution preparation.

First, the ground slag with a specific amount was added to the predetermined amount of solvent dimethylformamide (DMF) and sonicated for 1 h to get a well-dispersed solution. The precalculated amount of the waste polymer was added, and the solution was stirred at 50 °C for 3 h. For the SDS-modified membrane, SDS was added to the PS/Slag/DMF solution and stirred for 24 h at room temperature to get the resultant solution ready for casting and cloud point determination. Hereinafter, the dope solution was cast manually on a glass plate using 200 μm stainless steel blades, coagulated in a water bath for half an hour, and finally oven-dried at 40 °C, Figure 1, to get the final flat sheet asymmetric membranes with a skin layer morphology.

#### Binodal Determination

The structural characteristics of both symmetric and asymmetric membranes produced through precipitation can be explained by considering thermodynamic and kinetic factors. These factors include the thermodynamic impact (relating to thermodynamic effects) and the kinetic aspect (governing the duration of separation of the cast solution film when immersed in the nonsolvent bath from the glass plate). Cloud point determination was performed to track the doping solution behavior through the phase separation process. The previously mentioned solutions were titrated at room temperature with distilled water. Incremental additions of water were made to the solution using a micropipet with vigorous stirring. The titration end point was identified by the appearance of the solid phase. However, due to the dark milky color resulting from slag addition, the end point could not be visually or optically detected. Consequently, a series of solutions with specific concentrations of additives and polymers were prepared. After each addition of water, the solution was filtered. If no solid phase was observed, additional water was added to the subsequent solution, and this process was repeated until the solid phase formed.^26−30^

The steel slag and SDS amounts were manipulated as follows: Different amounts of steel slag were added (0.1–1.5 wt %) to 35% waste PS and the amount of water until reaching the cloud point was recorded with each concentration used. The same was done with SDS (0.1–1.5 wt % DMF) to figure out the effect of SDS addition, and finally, the optimized values of slag and SDS identified at which the amount of water stabilized.

**Figure 2:**
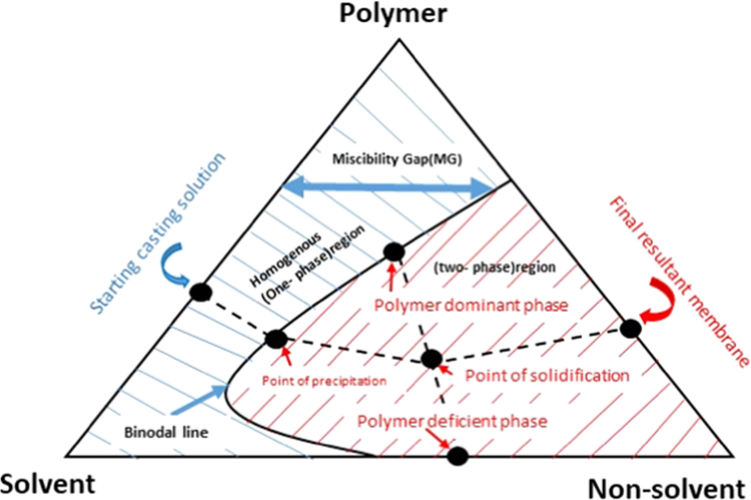
Tertiary phase diagram of the nonsolvent phase inversion method.

The utilization of a phase diagram facilitated the thermodynamic discussion regarding the membranes’ morphology. By water titration for the final casting dope solution, the cloud point is determined, at which point the polymer starts to precipitate out of the dope solution. The binodal line for different dope compositions could be utilized, as shown in Figure 2, as an indication of the thermodynamic instability of the solution. The thermodynamic instability predicts the final morphology of the obtained membranes, shows the effect of additives in the casting solution, and showcases the morphology changes, which affect the final characteristics of the obtained membranes.^26,31^ Different composite dope solutions at different PS waste concentrations were prepared whether with plain waste PS or with the optimized values of slag and slag with SDS to show the effect of additives on the amount of water and binodal line position and, hence, the thermodynamic instability.

The thermodynamic performance of the prepared solutions was reviewed by determining the degree of shift in binodal line (DSBC) in terms of the length of miscibility gap (MG) with and without additives by using eq 1(26)
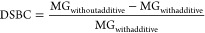
1Then, we calculate *T*, a dimensionless parameter, to examine how the thermodynamic properties of the casting solution impact the ultimate morphology of the membrane, as described by eq 2(26)
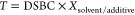
2where the interaction between the solvent and additive, denoted as *X*_solvent/additive_, is computed by eqs 3and 4(26)
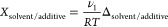
3
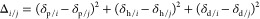
4In the given equation, δ denotes the solubility parameter, and δ_p_, δ_h_, and δ_d_ represent dispersive, hydrogen bond, and polar interactions, respectively. ν_1_ corresponds to the molar volume of the solvent (cm^3^/mol).

A preliminary estimate of the kinetics of phase separation can be readily acquired by assessing the viscosity of the casting solution.^28^ The kinetic behavior of the assembled solutions was tracked using viscosity measurements using the rheometer at shear rate (10 s^–1^).^32^ The membranes were cast using a doctor blade casting knife of a thickness (200 μm), put into a coagulation bath filled with water for 30 min, and dried for 24 h at 40 °C.

#### Membrane Characterization and Evaluation

For membrane characterization, FTIR was employed to confirm the incorporation of additives (steel slag or steel slag/surfactant) into the polymer matrix.^33^ The surface and cross-sectional morphology of the membranes were investigated by scanning electron microscopy (SEM; JCM-6000PLUS, Tokyo, Japan) after being coated with Pt/Pd alloy using a JEOL, JEC-3000FC, AUTO FINE COATER, for 75 s at 40 mA under 3 Pa for good resolution. ImageJ software was utilized to evaluate the mean pore size and distribution of the membranes.^34^ The hydrophobicity of the resultant membrane surfaces was demonstrated through contact angle (CA) measurements using the sessile drop method.^35^ A Vernier caliper was used for thickness measurement, by which the membrane thickness was determined at five different locations, and the average was recorded.

**Figure 3:**
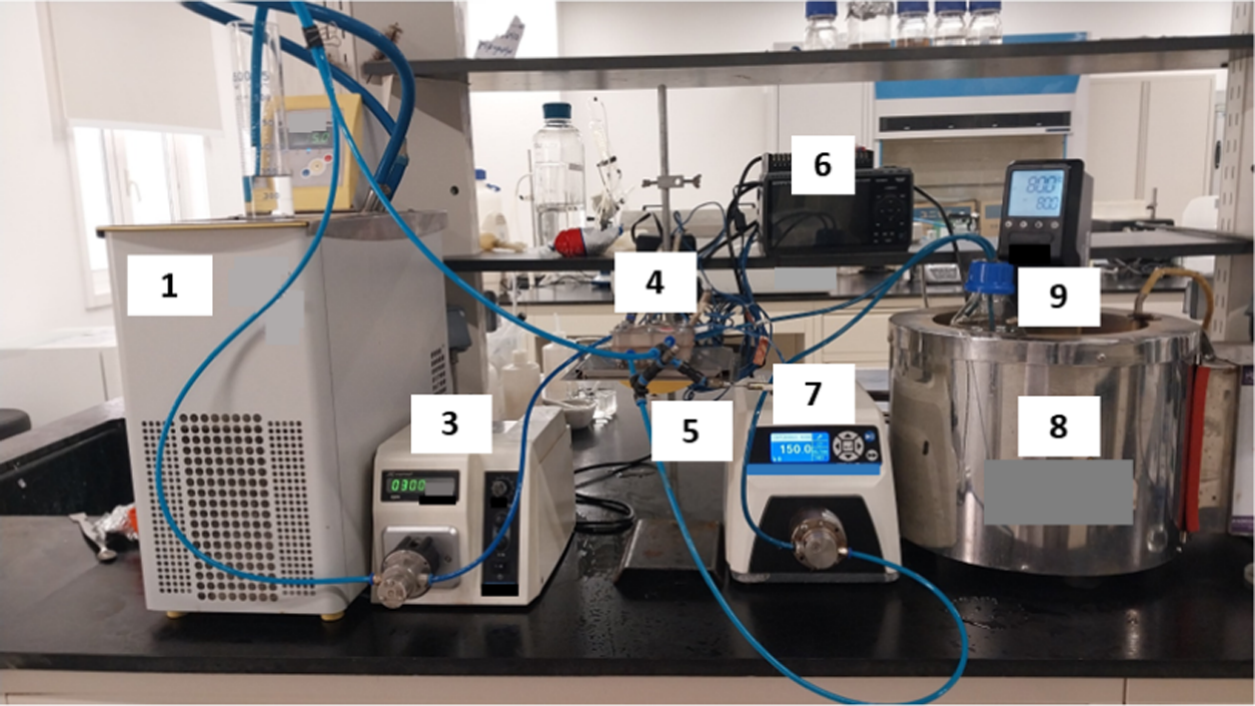
Distillation unit components: (1) chiller, (2) permeate container, (3) permeate pump, (4) MD cell, (5) thermocouples, (6) data logger, (7) feed pump, (8) feed heater, and (9) feed container.

Surface porosity was calculated using a wet–dry gravimetric approach,^36^ where each membrane was immersed in isopropanol (α chemical, >99%) for 24 h, weighed after excess alcohol was removed (WW), and then dried at 40 °C for 24 h and reweighed (WD). The surface porosity of each membrane was calculated using eq 5,^26^ which considers the weights of dry and wet specimens (WD and WW, respectively), the density of isopropanol at ambient temperature (*ρ*_IPA_), cross section of the tested specimen (*A*), and its thickness (*L*).
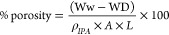
5Finally, for assessing the membrane performance, the composite membrane was tested for productivity using a customized membrane distillation system, and the PVDF commercial membrane was tested for comparison. A handmade direct contact membrane distillation (DCMD) unit was developed to test the performance of the obtained composite membrane. The experimental arrangement, as depicted in Figure 3, included a DCMD cell (constructed from acrylic with dimensions of 5 × 5 cm^2^), feed and permeate pumps, a chiller, a water bath (for heating the feedwater), thermocouples, a data logger, and associated connections.

## Results and Discussion

### Raw Material Characterization

**Figure 4:**
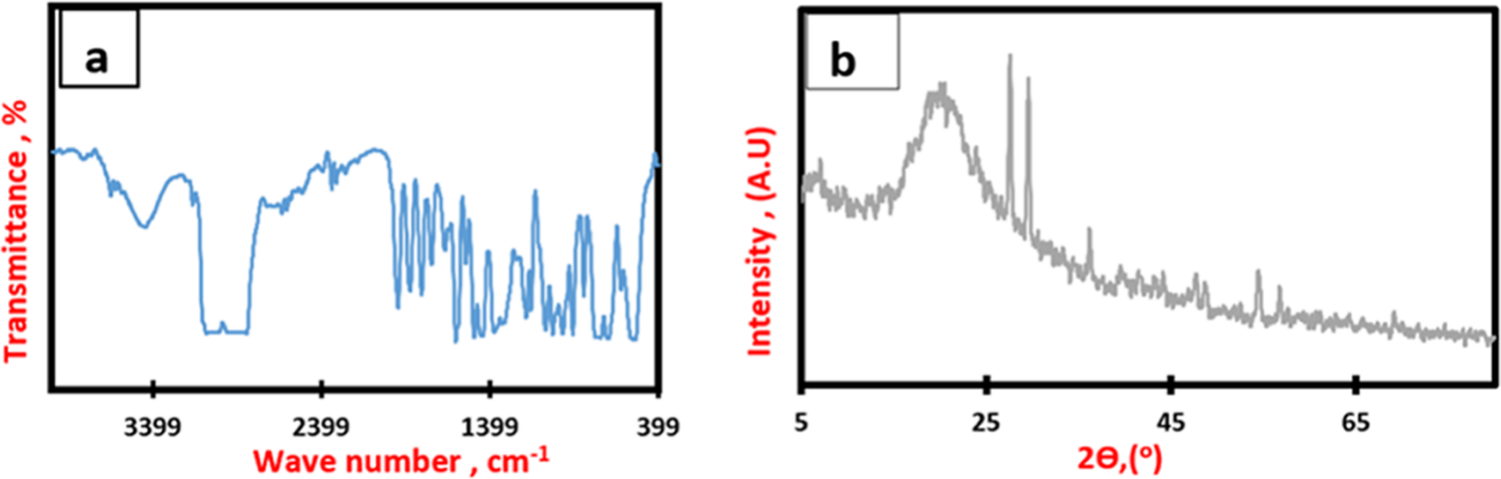
(a) FTIR of plain waste PS. (b) XRD of plain waste PS.

The main infrared peaks for the waste polystyrene, shown in Figure 4a, confirmed the presence of the HIPS type of waste. The peaks at 748, 1944, and 1600 cm^–1^ indicate the existence of an aromatic monosubstituted benzene ring.^37^ The peak at approximately 754 cm^–1^ is associated with *cis*-1,4 polybutadiene, while the peaks at 979–964 cm^–1^ correspond to C–C torsion and =C–H in-plane deformation of butadiene, indicative of the presence of polybutadiene.^3^ The peaks at 1451–1493 cm^–1^ indicated the deformation of CH_2_ + C=C of the aromatic ring. The aliphatic C–H and asymmetric stretch of CH_2_ could be approved by the peak at 2922.6 cm^–1^. The peak at 3060 cm^–1^ corresponded to the aromatic C–H stretching.^3^ In addition, the presence of silica additive was supported by the peaks at 1248, 1096, and 880 cm^–1^, which correspond to Si–CH_2_–R, Si–O–C bond, and the stretching vibrations of the Si–O bond, respectively.^38^

The X-ray diffraction (XRD) data is depicted in Figure 4b, covering the entirety of the utilized PS material. The results indicated the presence of relatively wide peaks, suggesting that the materials used have a semiamorphous nature. Furthermore, it confirms that PS is the primary component and silica serves as the principal additive in this context.^39^

**Figure 5:**
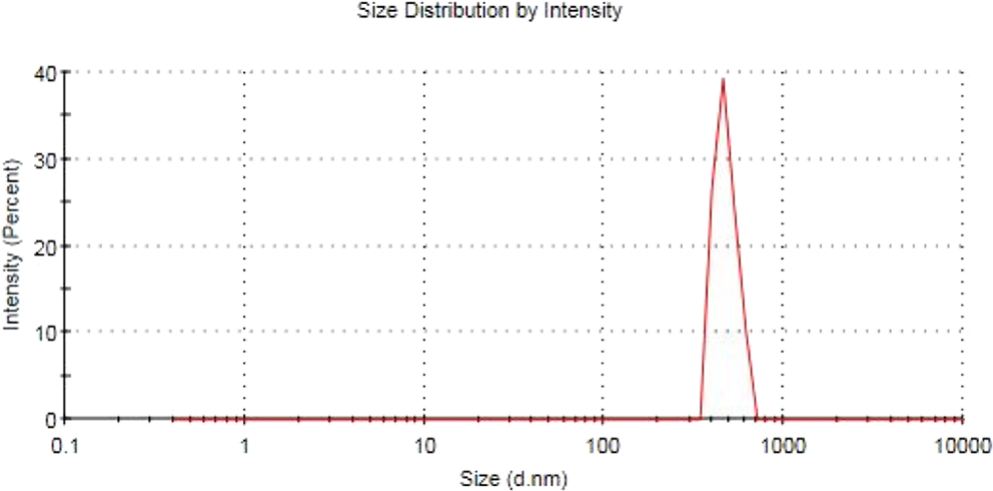
Zetasizer analysis of the used steel slag.

The X-ray fluorescence (XRF) analysis shown in Table 1 illustrated that the primary elemental composition of the EAF slag consisted of iron oxides, calcium oxides, silica, and a minor quantity of TiO_2_, accounting for 35.5, 45.5, 9.09, and 0.446%, respectively. Additionally, there were 1.824% by weight of other elements that were attributed to loss on ignition (LOI). Particle size analysis of the used slag particles has been performed using a Zetasizer. Figure 5 shows slag particles’ size of 475.5 nm.

**Table 1:** XRF of the Steel Slag

element	SiO_2_	Fe_2_O_3_	Cao	MgO	Al_2_O_3_	MnO	TiO_2_	LOI
%	9.09	35.5	45.5	2.09	3.03	2.52	0.446	1.824

### Binodal Graphs

**Figure 6:**
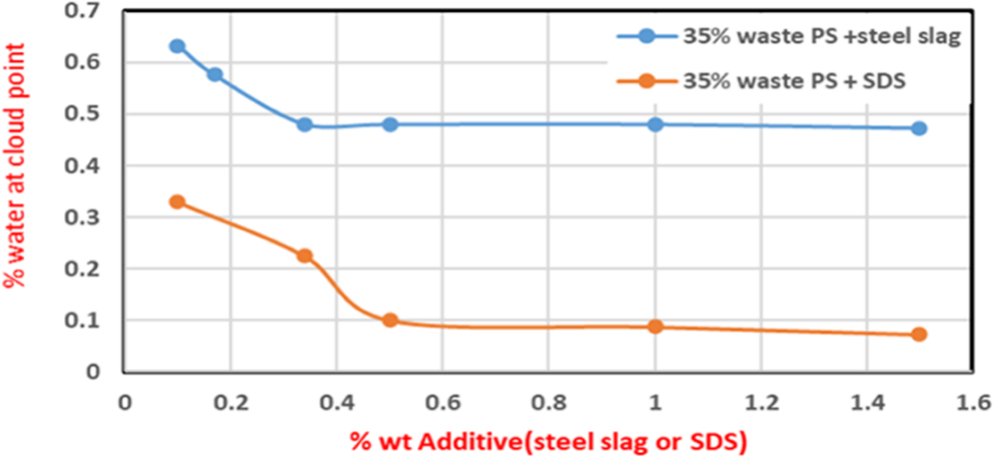
Water content variation with additives at a fixed polymer concentration of 35 wt %.

As aforementioned, different amounts of steel slag were added (0.1–1.5 wt %) to 35% waste PS, and the amount of water until reaching the cloud point was recorded. When steel slag was added in ascending order, the amount of water declined by more than 30% (∼31%) and retained its value at 0.3 wt % steel slag. While SDS was utilized, the water amount reduction was more pronounced as it largely declined by ∼90%. Further addition of SDS retained the value at 0.5 wt % SDS relative to DMF. The results, as shown in Figure 6, illustrated that the optimum amounts of additives were assigned to the point on which the water content of coagulation remains constant for both additives, 0.3 wt % solution for steel slag and 0.5% DMF for SDS.

The ternary phase diagrams of waste HIPS/DMF/water systems constructed by cloud point measurements showed discrepancies in the binodal lines among the different prepared composites.

**Figure 7:**
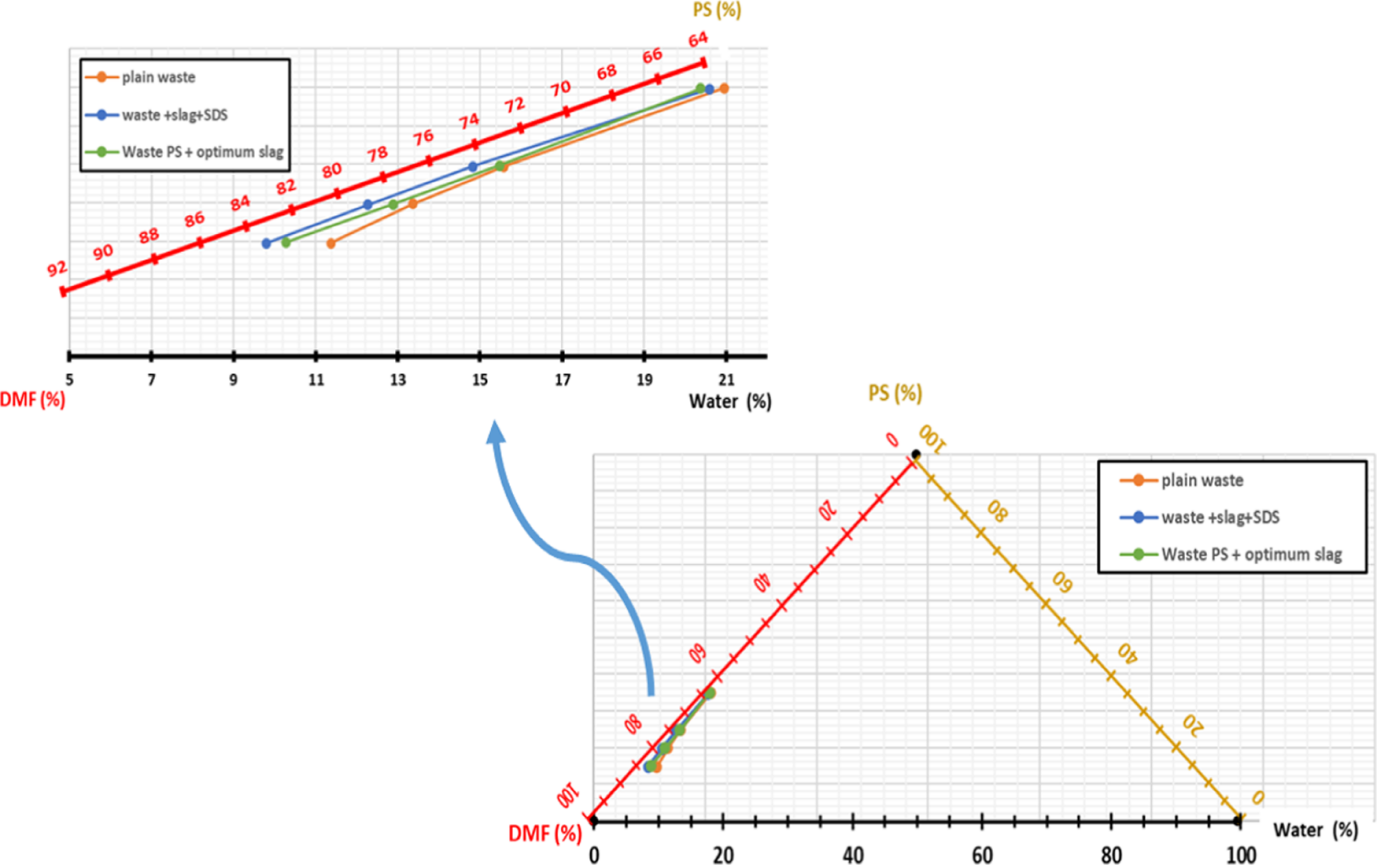
Ternary phase diagram for different prepared membranes with binodal curves.

From the ternary phase diagram, Figure 7, the miscibility gap (MG) was calculated from which the degree of shift of the binodal curve was evaluated using eq 1. By using the Hansen solubility parameters of the different components, Table 2, the thermodynamic parameter *T* was calculated using eqs 2–4, and the values are given in Table 3. The ratio of Δsolvent/additive (Δ*i*/*j*) is calculated by eq 4 from Hansen solubility parameters of components, as given in Table 2.

**Table 2:** Hansen Solubility Parameters of Different Components

components	δ_p_ (MPa)^1/2^	δ_h_ (MPa)^1/2^	δ_d_ (MPa)^1/2^	molar volume (cm^3^/mol)
PS^20,40,41^	4.5	2.9	18.5	11.69
Fe_2_O_3_^41^	12.3	14.3	20.7	63.02
DMF^41^	13.7	11.3	17.4	77.0
Water^41^	16.0	42.3	15.6	18
SDS^41^	15.0	8.0	14.0	285.49

**Table 3:** Thermodynamic Parameters of Different Prepared Membranes

type of membrane	MG (inch)	DSBC	DSBC (%)	*T*
15% waste	1.00	0.00		
15% waste + 0.3% slag	0.63	0.37	37.00	189.12
15% waste + 0.3% slag + 0.5% SDS	0.47	0.53	53.00	307.58
35% waste	0.66			
35% waste + 0.3% slag	0.58	0.12	12.12	61.96
35% waste + 0.3% slag + 0.5% SDS	0.51	0.23	22.73	131.89

The parameters Δ_DMF/Fe_2_O_3__, Δ_DMF/SDS_, and Δ_DMF/PS_ were revealed as follows: 21.85, 24.14, and 156.41 MPa, respectively. Lower Δ*i*/*j* induces greater affinity between two components, which indicates the enhanced affinity and solubility of the polymer mixture by additives.

As presented in the results, by adding the slag to the PS waste, the binodal line moved toward the polymer–solvent (PS-DMF) axis, and the homogeneous region was decreased. As a result, a reduced amount of water is required for the precipitation of the casting solution, leading to an increased likelihood of demixing. Same with adding the SDS to the PS, the water amount needed for precipitation decreases, implying the enhancement of thermodynamic instability. Moreover, adding the surfactant in the presence of slag largely increased the thermodynamic instability and enhanced the demixing process. The DSBC for slag alone and slag with SDS increased to 12 and 23%, respectively, at 35% waste PS concentration. The effect is even pronounced with a decreasing amount of waste hitting (53% increase) at 15% PS concentration.

The viscosity of 35% PS concentration dope solutions was measured as shown in Table 4, and adding slag and SDS reduced the viscosity of the casting dope solution by 62.49 and 71.34%, respectively. The additives enhanced the kinetics and accelerated the mass transfer between the solvent and nonsolvent in the coagulation bath. Due to the diminished viscosity of the casting solution, water diffused more quickly into the PS with steel slag and SDS in the coagulation bath. This accelerated diffusion, in turn, facilitated the creation of macro voids, resulting in the formation of more porous membranes with larger cavities in the sublayer compared to membranes formed without additional components.^4,31^

**Table 4:** Shear Viscosity at a 10 s^–1^ Shear Rate for 35% PS Concentration Membranes

membrane type	shear viscosity (Pa·s)	standard deviation
35% waste	8.234	1.394
35% waste + 0.3% slag	3.282	0.476
35% waste + 0.5% SDS	2.887	0.023
35% waste + 0.3% slag + 0.5% SDS	2.245	0.188

### Membrane Characterization and Evaluation

**Figure 8:**
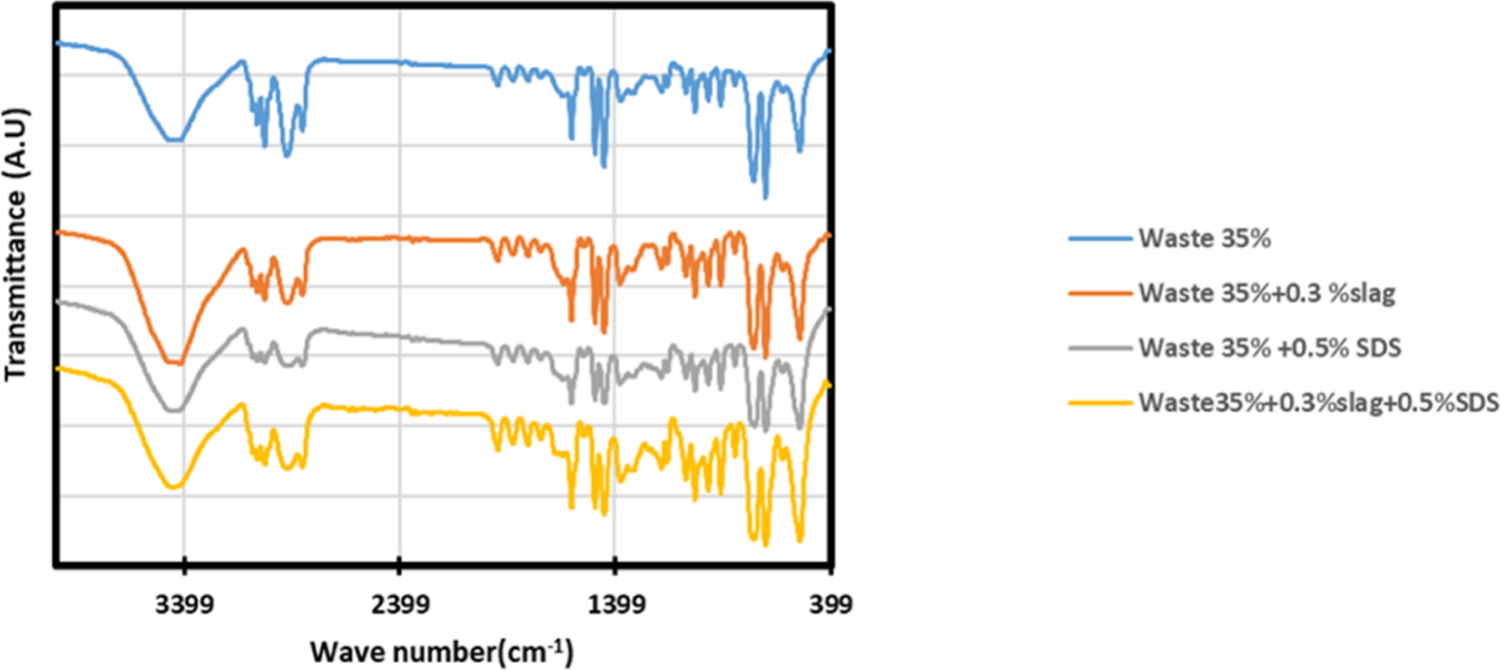
FTIR of the prepared 35% waste HIPS membrane.

FTIR has been utilized to characterize the chemical composition of the obtained 35% PS concentration membranes and ensure the successful additive inherence within the obtained polymer matrix (Figure 8). For the whole tested membranes, the assured existence of HIPS is determined by the peaks observed at 2917.18, 3082, and 3026 cm^–1^ that are attributed to the stretching vibrations of C–H bonds. Additionally, the absorption peaks at 1595.81 and 1446.59 cm^–1^ were associated with the vibrations of aromatic C=C bonds.^22^ The absorption bands at 738, 911 cm^–1^ are characteristics of 1,4, 1,2 *cis* and vinyl units of polybutadiene.^42^ Furthermore, the addition of steel slag appears as intense peaks at 518.89 and 1487.49 cm^–1^, representing the vibration bond of Fe–O, Mg–O, Ti–O, and other metal oxides, as well as CaO.^13^ SDS existence appears as distinct bands at 3467–2871 cm^–1^^43^ and there are four distinctive bands (within the 900–1600 cm^–1^ range), with 993, 1219, and 1278 cm^–1^ linked to the vibrational modes of the sulfate group (S=O stretching vibration) and 1472 cm^–1^, corresponding to the scissoring of the methylene group.^44^ The presence of additives originated from waste PS and slag appeared as peaks of silicon were detected by bands associated with the long-chain Si–CH_2_–R groups, specifically at 1248 cm^–1^, along with another band at 1096 cm^–1^ assigned to the Si–O–C bond. Additionally, a band linked to the stretching vibrations of the Si–O bond was observed at 880 and 1001.91 cm^–1^. Furthermore, the bending vibration bond at 709 cm^–1^ is indicative of Si–O–Al.^22^ The broad band centered around 3436 cm^–1^ observed in all samples, along with the band at 1638 cm^–1^, is likely attributed to the bending vibration of −OH.^45−48^

**Figure 9:**
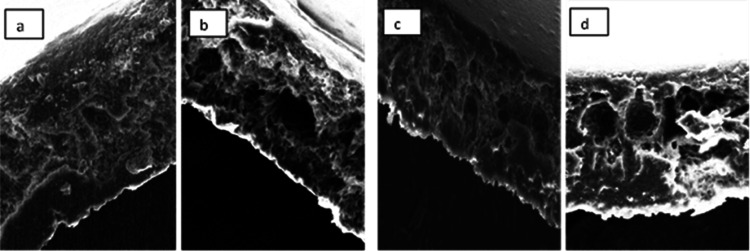
FESEM cross-sectional images: (a) 35% plain waste PS, (b) 35% waste PS + 0.3 wt % steel slag, (c) 35% waste PS + 0.5 wt % SDS, and (d) 35% waste PS + 0.3% steel slag + 0.5 wt % SDS.

The field emission scanning electron microscopy (FESEM) cross-sectional images of the membranes are shown in Figure 9a–d. All membranes showed an asymmetric cross-sectional structure with a porous sponge-like structure, implying relatively slow kinetics precipitation rates. However, the addition of additives, such as steel slag or SDS, improved the morphology to macro void formation and enhanced demixing, as proved from cloud point determination. Adding slag and SDS decreased the nonsolvent tolerance of the polymer solution. This caused instantaneous demixing and the formation of a more finger-like structure with a thinner skin layer, showing the enhanced demixing process and precipitation rate during the phase inversion process. At higher demixing rates, membranes with large macro voids produced.^28^

Furthermore, porosity measurements, Table 5, confirmed the morphology enhancement through demixing and the high kinetics rate. By adding the steel slag to the polymer solution, porosity slightly increased, with a rise value of 43.22%. Moreover, adding both the SDS and slag leads to more than 25% porosity increase.

**Table 5:** Porosity Values of the Prepared Membranes

type	PVDF commercial	35% plain waste	35% + 0.3% slag	35% + 0.5% SDS	35% + 0.3% slag + 0.5% SDS
porosity (%)	61.666	36.922	36.950	43.427	46.260
standard deviation	2.624	0.446	0.515	0.895	0.465

**Figure 10:**
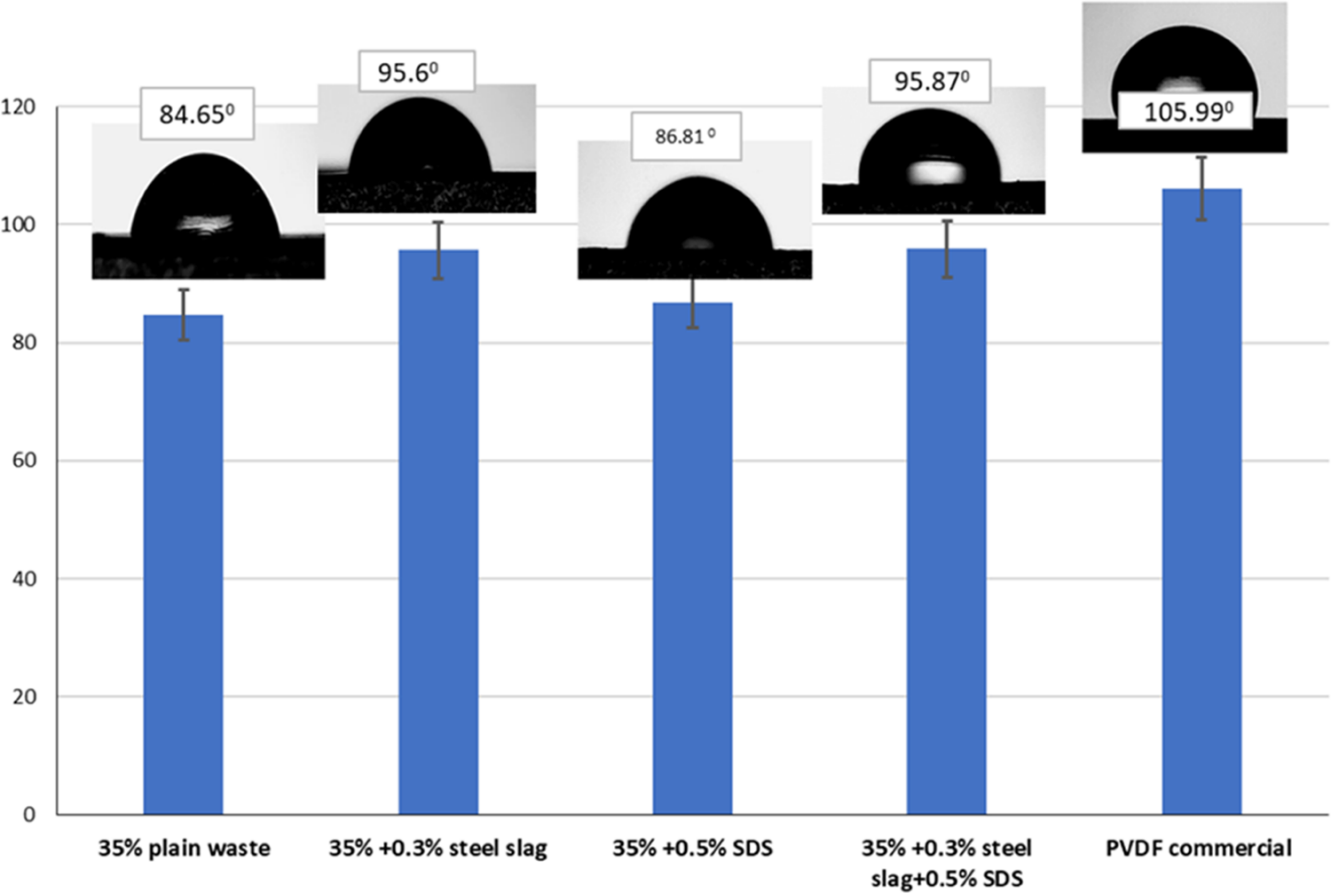
Contact angle measurements of the prepared membranes.

The hydrophobicity of the membranes was assessed by measuring the contact angle between a water droplet and the membrane surface; the results are illustrated in Figure 10.

This determination was made through the measurement of contact angles with droplets of distilled water. It could be interpreted that introducing the steel slag and SDS to the casting solution slightly increased the surface roughness of the obtained membranes and hence slightly increased the contact angle of the membranes^49,50^ and that the effect was more pronounced for the steel slag more than the SDS alone while the mix of the two enhanced the hydrophobicity by 13% to be 95.87°, and these values were comparable to the commercial PVDF one.^51^

Finally, the performance testing of the composite membrane in DCMD cell is presented for productivity investigation in Table 6. It showed that the prepared membrane gave a permeate flux of (1.043 kg/m^2^·h) compared with that (4.250 kg/m^2^·h) of commercial PVDF membrane. Although the flux is relatively low, the same flux could be attained with a larger area of the composite membrane with around 99% reduction in the raw material cost. Compared to the literature, Table 7, these results made the prepared composite competitive among the others prepared before.

**Table 6:** MD Testing Results^a^

membrane type	membrane features	operating conditions	flux (kg/m^2^·h)	standard deviation
PVDF commercial	θ = 73.47°, ε = 61.675%	NaCl: 1 wt %; *T*_f_: 64 °C; *T*_p_: 21 °C; MA: 5 cm × 5 cm; *Q*_f_: 100 mL/min; *Q*_p_: 100 mL/min	4.250	0.409
composite (HIPS/slag/SDS membrane)	θ = 95.87°, ε = 46.26%		1.043	0.077

a θ: water contact angle; ε: porosity; *T*_f_: inlet feed temperature; *T*_p_: permeate feed temperature; MA: membrane area; *Q*_f_: feed flow rate; *Q*_p_: permeate flow rate.

**Table 7:** Performance Comparison of the Composite Membrane with the Literature

membrane type	flux (kg/m^2^·h)	references
PTFE-PP supported	1.8–10	(52)
PP S6/2	0.5–6	(53)
PP (hollow fiber)	0.7–4.2	(54)
GVHP (flat sheet membrane)	2.988	(55)
MD080CO2N (capillary and hollow fiber)	2.988	(56)
PVDF 93-2 (hollow fiber)	2.621	(57)
PVDF (hollow fiber)	0.1–1.95	(58)
PVDF supported flat sheet	0.02–1.73	(59)
Modified CA (modified flat sheet)	1.368	(60)
CO17 (hollow fiber)	1.296	(61)
Casted PS/slag composite (flat sheet)	0.997	This work
PVDF 43-1 uncoated (hollow fiber)	0.878	(62)
PE (hollow fiber)	0.864	(54)
PVDF 43-1 (Si-LTV coated) (hollow fiber)	0.284	(62)
PVDF (hollow fiber)	0.036–0.576	(63)

## Conclusions

Preparation of a waste HIPS/slag/SDS composite polymeric membrane in aid of SDS as the surfactant using the solution-casting method was attained. The thermodynamic stability investigation was attained by ternary phase diagram construction using a cloud point determination. The results showed shifts of the binodal line toward the solvent–polymer axis and reduced miscibility gap for all prepared membranes in the specified range using steel and SDS as additives. Within the studied range of (0.1–1.5%) for both additives, both the thermodynamic instability and mass transfer between the solvent, additives, and nonsolvent enhanced introducing a more porous structure with increased porosity up to 25% increase and enhanced hydrophobicity with contact angle reaching ∼96°. This enhanced recycled cost-effective composite membrane out of waste materials gave a flux of 1.043 kg/m^2^·h with around 99% raw material cost reduction, making the membrane a candidate for water desalination using DCMD.

## Data Availability

All the data supporting the findings of the study are available in the article.

## Supporting Information Available

Experimental data error for shear viscosity (Table S1); porosity (Table S2); and permeate flux (Table S3) (PDF)
