# Supplementary material for: Morphology and Thermodynamic Study of a Novel Composite Membrane from Waste Polystyrene/Slag: Experimental Investigation
Source: ACS Omega. 2024 May 21;9(22):23512–22. doi: 10.1021/acsomega.4c00671 (PMC11154918; doi:10.1021/acsomega.4c00671)
Supplement: Supplementary file 1 — ao4c00671_si_001.pdf [file ao4c00671_si_001.pdf]

Supporting Information

Morphology and Thermodynamic Study of Novel Composite Membrane from Waste Polystyrene/Slag: Experimental Investigation

Salma Tarek Ghaly [1,2] **, Usama Nour Eldemerdash*[1,3]*, and Ahmed Hassan El-Shazly* [1,4]

[1]Chemical and Petrochemical Engineering Department, Egypt-Japan University of Science and Technology, New Borg AL Arab city, Alexandria, Egypt.

[2]Central Metallurgical Research and Development Institute (CMRDI), PO Box 87 Helwan, Cairo, Egypt.

[3]Benha Faculty of Engineering, Benha University, Qaliobiya, Egypt.

[4]Chemical Engineering Department, Faculty of Engineering, Alexandria University, Alexandria, Egypt.

KEYWORDS: Waste polystyrene, slag, membrane preparation, cloud point determination, ternary phase diagram, Membrane Distillation.

Table of Contents

Table S1: Experimental data error for shear viscosity S3

Figure S1: Experimental error for shear viscosity measurements S3

Table S2: Experimental data error for porosity S4

Figure S2: Experimental error for porosity measurements S4

Table S3: Experimental data error for permeate flux S4

Table S1: Experimental data error for shear viscosity

	35% waste	35% waste +0.340% slag	35% waste +0.5% SDS	35% waste trans +0.340% slag+0.5% SDS
	10.17	3.814	2.915	2.407
	8.888	3.553	2.901	2.233
	8.228	3.445	2.89	2.426
	7.294	2.9665	2.876	2.197
	6.588	2.631	2.854	1.962
**Mean**	8.234	3.282	2.887	2.245
**standard deviation**	1.393625057	0.476018697	0.023445682	0.18811034
**Coefficient of variance (COV)**	16.92607191	14.50436324	0.812056037	8.379079731

Figure S1: Experimental error for shear viscosity measurements

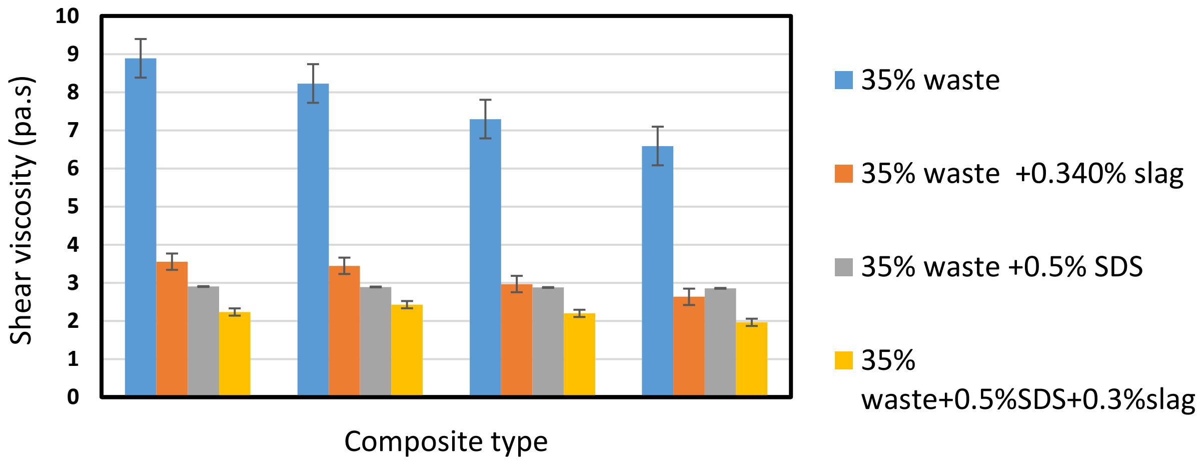

Table S2: Experimental data error for porosity

	PVDF comm	waste 35%	waste 35% +0.340% slag	waste 35% +0.5% SDS	waste 35% + 0.340% slag+0.5% SDS
	60.273	36.614	36.262	43.225	45.756
	64.153	36.545	37.713	42.446	46.045
	64.186	36.860	36.923	43.153	47.009
	58.051	37.668	36.900	44.884	46.229
**Average**	61.666	36.922	36.950	43.427	46.260
**standard Deviation**	2.624	0.446	0.515	0.895	0.465
**COV (coefficient of variance)**	4.256	1.209	1.393	2.060	1.004

Figure S2: Experimental error for porosity measurements

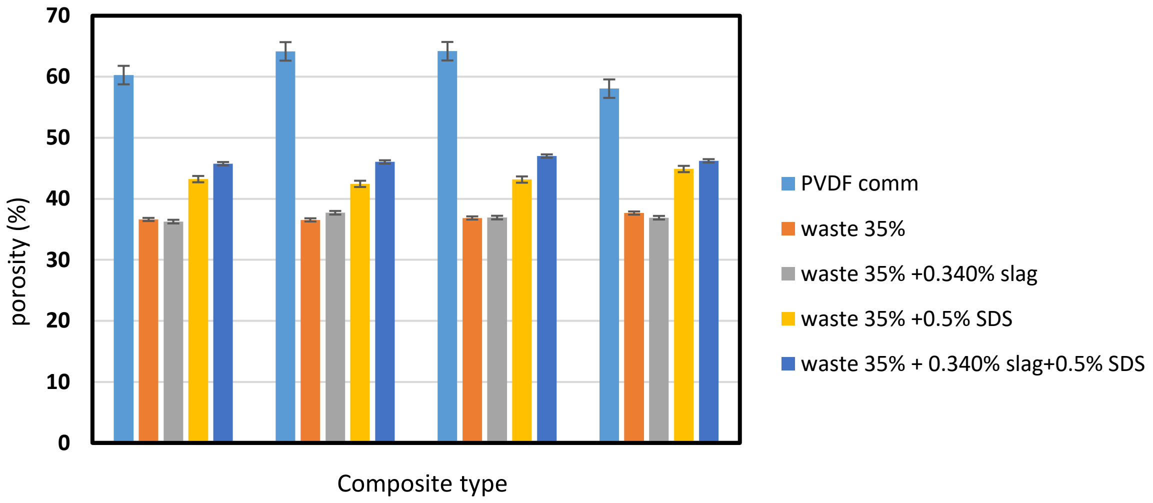

Table S3: Experimental data error for permeate flux

	waste trans35%+0.3% slag +0.5% SDS	PVDF membrane
	flux(kg/m^2.hr)	flux(kg/m^2.hr)
	1.137	4.800
	0.947	3.821
	1.047	4.129
**Average**	1.043	4.250
**Standard Deviation**	0.077	0.409
**Coefficient of variance (COV)**	7.413	9.619
